# Coccidioides Species: A Review of Basic Research: 2022

**DOI:** 10.3390/jof8080859

**Published:** 2022-08-16

**Authors:** Theo N. Kirkland, David A. Stevens, Chiung-Yu Hung, Sinem Beyhan, John W. Taylor, Lisa F. Shubitz, Sascha H. Duttke, Arash Heidari, Royce H. Johnson, Stanley C. Deresinski, Antje Lauer, Joshua Fierer

**Affiliations:** 1 Department of Medicine, Division of Infectious Disease, San Diego School of Medicine, University of California, San Diego, CA 92093, USA; 2 Department of Pathology, San Diego School of Medicine, University of California, San Diego, CA 92093, USA; 3 California Institute for Medical Research, San Jose, CA 95128, USA; 4 Division of Infectious Diseases and Geographic Medicine, Department of Medicine, Stanford University Medical School, Stanford, CA 94305, USA; 5 South Texas Center for Emerging Infectious Disease, Department of Molecular Microbiology and Immunology, University of Texas at San Antonio, San Antonio, TX 78249, USA; 6 Department of Infectious Diseases, J. Craig Venter Institute, San Diego, CA 92037, USA; 7 Department of Plant and Microbial Biology, University of California, Berkeley, CA 94720, USA; 8 Valley Fever Center for Excellence, University of Arizona, Tucson, AZ 85724, USA; 9 School of Molecular Biosciences, College of Veterinary Medicine, Washington State University, Pullman, WA 99164, USA; 10 Department of Medicine, Division of Infectious Diseases, Kern Medical, Bakersfield, CA 93306, USA; 11 Valley Fever Institute, Bakersfield, CA 93306, USA; 12 Department of Medicine, David Geffen School of Medicine UCLA, Los Angeles, CA 90095, USA; 13 Department of Biology, California State University Bakersfield, 9001 Stockdale Highway, Bakersfield, CA 93311, USA; 14 Infectious Diseases Section, VA Healthcare San Diego, San Diego, CA 92161, USA

**Keywords:** *Coccidioides immitis*, *Coccidioides posadasii*, coccidioidomycosis, fungus, dimorphic fungus, mycelium, spherule, mycology, microbiology, pathogenesis

## Abstract

*Coccidioides immitis* and *posadasii* are closely related fungal species that cause coccidioidomycosis. These dimorphic organisms cause disease in immunocompetent as well as immunocompromised individuals and as much as 40% of the population is infected in the endemic area. Although most infections resolve spontaneously, the infection can be prolonged and, in some instances, fatal. *Coccidioides* has been studied for more than 100 years and many aspects of the organism and the disease it causes have been investigated. There are over 500 manuscripts concerning *Coccidioides* (excluding clinical articles) referenced in PubMed over the past 50 years, so there is a large body of evidence to review. We reviewed the most accurate and informative basic research studies of these fungi including some seminal older studies as well as an extensive review of current research. This is an attempt to gather the most important basic research studies about this fungus into one publication. To focus this review, we will discuss the mycology of the organism exclusively rather than the studies of the host response or clinical studies. We hope that this review will be a useful resource to those interested in *Coccidioides* and coccidioidomycosis.

## 1. Introduction

Coccidioidomycosis is an important pathogenic fungal infection in much of the desert and semi-arid regions of the Western hemisphere. The incidence of the disease is increasing in both California and Arizona [1,2]. Although not all infections result in clinical illness, the infection frequently causes serious disease in immunocompetent people and even self-limited coccidioidomycosis can be a prolonged illness requiring anti-fungal therapy. Coccidioidomycosis is a common cause of community-acquired pneumonia in the endemic area that is frequently misdiagnosed, and the cost of the disease is substantial [3,4]. In patients where the infection is not self-limited, the disease can spread to skin, bone, and the central nervous system among many other organs. Although disseminated disease is unusual, it occurs in both immunocompetent and immunocompromised people, is frequently difficult to treat, and can be fatal. Prolonged treatment is usually required, and meningitis requires life-long therapy. For these reasons, a detailed understanding of the organism is important for improving our ability to diagnose and treat this illness. This is a detailed review of *Coccidioides* spp. fungi from a basic research perspective.

## 2. *Coccidioides* Spp.

### 2.1. Taxonomy

*Coccidioides immitis* and *C. posadasii* have been classified in a number of different ways over the years. In the 1890s *Coccidioides* was initially classified as a protozoan, and the growth of a mold from a lesion was thought to be a contaminant [5,6]. In 1905 Ophuls published a paper describing the pathology of the disease in detail, growing the organism, determining that it was a mold, and identifying spherules in human tissue and arthroconidia in mycelial culture [7]. In addition to these mammoth accomplishments, he inoculated guinea pigs and rabbits with the mold and showed they developed lesions identical to the human pathology. This clearly established that coccidioidomycosis was a fungal disease and made the era of “protozoa” misidentification a very brief one.

The subsequent classification of the fungus was another issue. Through the late twentieth century the primary pathogenic fungi (*Blastomyces* spp., *Coccidioides* spp., *Histoplasma* spp., and *Paracoccidioidomyces* spp.) were classified as Deuteromycotina or Fungi Imperfecti because, at that time, the sexual state of those human pathogens had not been identified and sexual morphology was necessary for classification in the Kingdom Fungi. The application of DNA variation to fungal taxonomy eliminated the need for sexual features in taxonomy, and among the first “asexual” fungi shown to belong to the fungal kingdom and the phylum Ascomycota were the primary pathogenic fungi, including *Coccidioides* [8]. Shortly thereafter, molecular taxonomy was applied to many pathogenic fungi, all of which could then be integrated into the Kingdom Fungi [9]. The order Onygenales was proposed and included all the dimorphic fungal species known at that time, which caused invasive diseases in humans as well as the dermatophytes [10,11]. Follow-up studies of a broader group of Onygenalean fungi by these and other investigators [8,11] showed that non-pathogens were interspersed with pathogens throughout the Onygenales and confirmed that *Uncinocarpus reesii* was closely related to *Coccidioides* spp.

In 2004, *Blastomyces* spp., *Histoplasma* spp., and *Paracoccidioidomyces* spp. were reclassified as Ajellomycetacae by DNA homology studies, but *Coccidioides* spp. did not fall into that group [12]. Instead, it was most closely related to a group of non-pathogenic organisms that fell between Ajellomycetacae and the Gymnoascaceae clade (which contains dermatophytes). Another study using slightly different techniques to determine DNA homology also found that Ajellomycetacae and Onygenaceae (containing *Coccidioides* spp. and a variety of non-pathogenic species) were sister groups [13].

**Figure 1:**
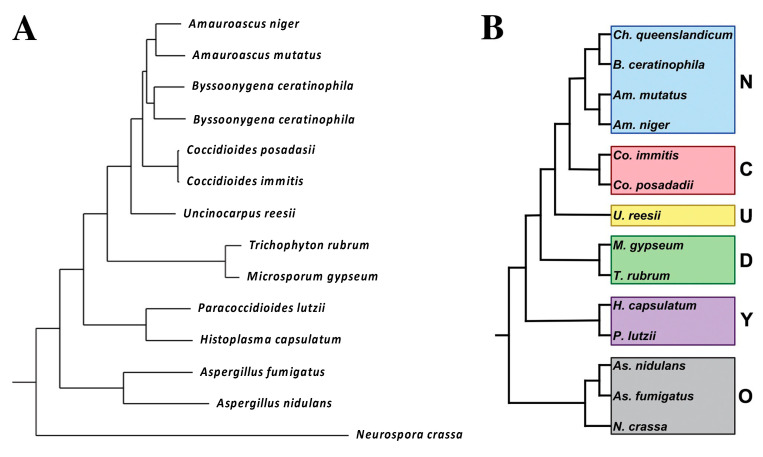
Phylogenetic distance tree (Bayesian) with posterior probabilities based on 100 randomly selected single-copy orthologs (**A**). Phylogenetic categories used for gene family expansion/contraction and ortholog group analysis (**B**). N, newly sequenced genomes. C, *Coccidioides*, U, *U*. *reesii*; D, dermatophytes; Y, yeast-forming dimorphic fungal pathogens; O, outgroups. The figure is from [14].

Whiston and Taylor took a more inclusive approach [14]. They reasoned that it is difficult to determine relatedness of *Coccidioides* spp. to non-pathogens when, among them, only the *U. reesii* genome had been sequenced. To address this, they determined the genome sequence of *Amauroascus niger, Amauroascus mutatus*, *Chrysosporium queenslandicum,* and *Byssoonygena creatinophila* and predicted the transcript assemblies/gene models [14]. A phylogenetic tree was built by choosing 100 single-copy orthologs that were present in all the species, concatenating and aligning them. The phylogenetic tree is shown in Figure 1.

The major conclusion of this study is that at least five non-pathogenic organisms were closely related to *Coccidioides* spp. A total of 791 genes were identified that were unique to *Coccidioides* spp. These genes tend to be up-regulated in spherules (compared to mycelia) [15,16], suggesting that they might be important for differentiation into the pathogenic spherule phase.

**Figure 2:**
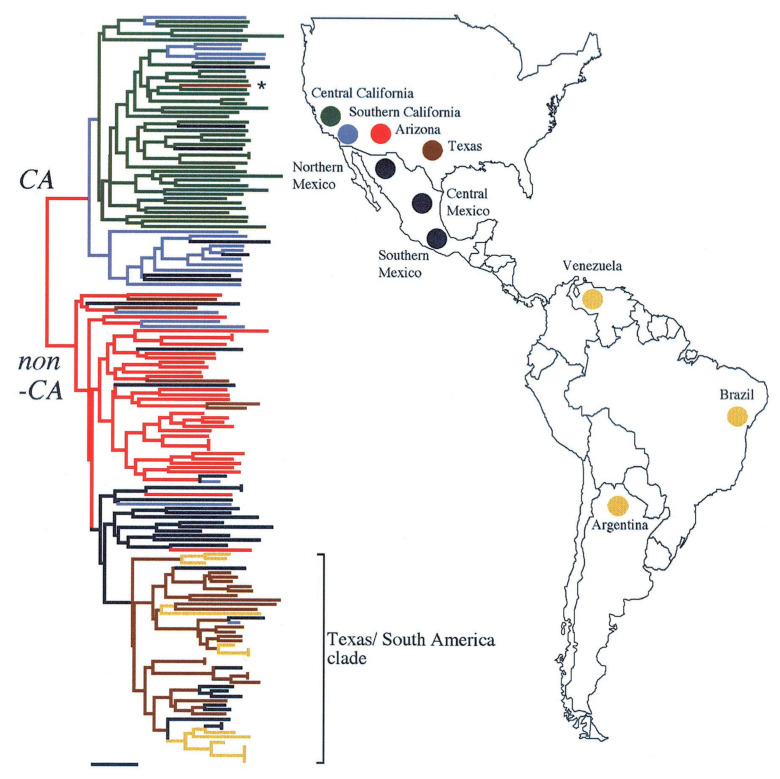
Geographic distribution of CA and non-CA strains. Biogeographic range expansion into South America by *C. immitis* mirrors New World patterns of human migration. The colors refer to the genetic clusters on the left. * means: The asterisk marks an isolate from a patient who was diagnosed in Texas but had acquired the disease in California. The figure is from [21].

More recently, *Coccidioides* spp. has been placed within the family Onygenaceae, based on the DNA homology of the PRP8 gene [17]. The only other pathogen in the Onygenaceae group was *Ophidiomyces ophiodiicola*, which is a pathogen of snakes. The phylogenetic tree from their study is shown in Figure 2. In this phylogeny, the family Onygenaceae is most closely related to the family Arthrodermataceae (containing the dermatomycetes), and both are slightly more distantly related to the family Ajellomycetaceae (containing the other systemic dimorphic pathogens *Histoplasma* spp., *Blastomyces* spp., and *Paracoccidioides* spp.); however, the statistical support for the basal branches determining these basal relationships is weak.

What is the overall conclusion from all this effort? Although thermally dimorphic human pathogens cause similar diseases, they seem to be more genetically distinct (polyphyletic) than once imagined. It appears that the ability of Onygenales to cause invasive human infections has evolved at least twice, once in the Ajellomycetacae (*Blastomyces* spp., *Histoplasma* spp., and *Paracoccidioides* spp.) and once again in the Onygenaceae (*Coccidioides* spp.). This also suggests that genetic programs underlying virulence in mammals may be significantly different in these two taxa and raises doubts about drawing conclusions based on homology between these human pathogens. The concept that thermal dimorphism and pathogenicity for humans has repeatedly evolved in *Coccidioides* spp., *Paracoccidioides* spp., *Histoplasma* spp., *Talaromyces marneffei,* and *Sporothrix* spp. has been previously discussed [18].

#### *C. Immitis* and *C. Posadasii*

Until 1996, *C. immitis* was thought to be the only species of *Coccidioides* and it was thought to be an asexual organism. In that year Taylor and colleagues reported evidence of recombination and the division of the isolates into two geographic groups, one in California and the other in Arizona, Texas, and Mexico [19,20]. These groups were initially referred to as CA and non-CA groups. Further studies of polymorphisms included many isolates from South America in the non-CA group, which proved to be more closely related to strains from Texas than strains from Arizona (Figure 2) [21].

**Figure 3:**
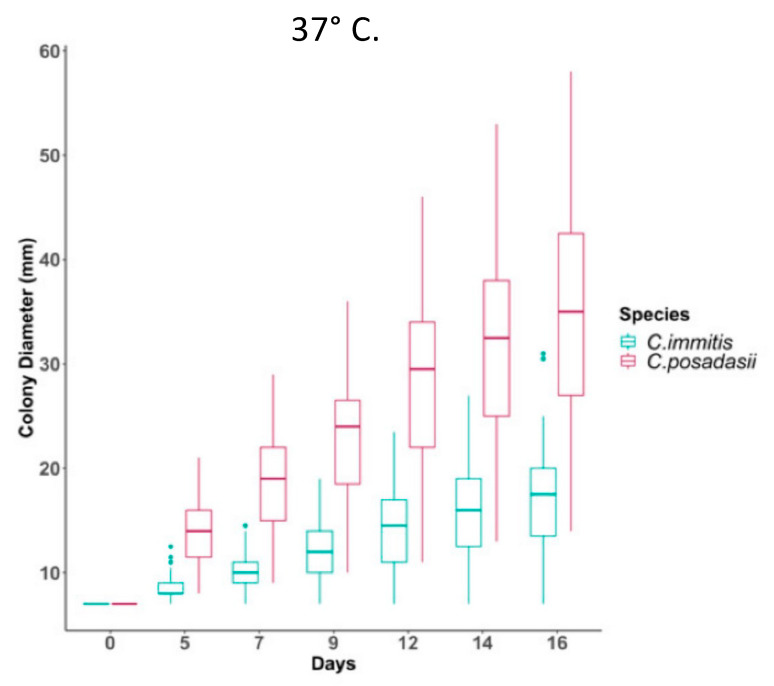
Temperature impacts growth ability of *C. immitis* isolates compared to *C. posadasii* on yeast extract media. Radial growth measurements at 37 °C for 46 *C. posadasii* and 39 *C. immitis* isolates in triplicate. The figure is from [23].

Fisher and colleagues estimated that the two taxa had been reproductively isolated about 8 million years ago, although this estimate was later revised downward. Eventually these two taxa were named *C. immitis* and *C. posadasii* [22]. *C. posadasii* was able to grow as mycelia on high salt media more rapidly than *C. immitis*. It has also recently been reported that *C. posadasii* mycelia grow more rapidly at 37 °C than *C. immitis* mycelia (Figure 3) [23].

Differences in temperature effects on spherule growth have not been reported. Genome sequencing has confirmed the two species are distinct with some interspecific gene flow. As far as we know, other major phenotypic differences between *C. immitis* and *C. posadasii* have not been reported, although that question has not been studied extensively. Certainly, serologic tests are cross-reactive and there is no evidence that protective immunity is species-specific. Since *Coccidioides* spp. usually are not speciated in the clinical laboratory, it is currently difficult to assess any differences in the clinical human disease produced by the two species. Nevertheless, further studies will no doubt reveal more phenotypic differences between the two species. One observation that bears further study is the inability of experienced researchers to cultivate *C. posadasii* from soil placed on growth media [24], whereas direct cultivation of *C. immitis* from soil has been routine for more than half a century [25]. It is possible that *C. posadasii* has evolved the ability to limit spore germination to mammalian lungs, thereby preventing germination in inhospitable environments, but no data have been published on this topic.

## 3. Geographic Distribution and Ecology

*Coccidioides* spp. are found in the desert and semi-arid soils of the Western hemisphere. The geographic limitations of these organisms were one of the first characteristics that were appreciated [26,27]. The rate of positive skin test reactions in human beings has been used to estimate the presence of the organism in the soil. One of the early studies of coccidioidin skin test reactivity in military recruits found that the skin test positive rate was over 50% in some areas of California, Arizona, and Texas [27]. The area of highest frequency in California was the San Joaquin Valley and in Arizona and Texas the highest rates were found in counties toward the south.

**Figure 4:**
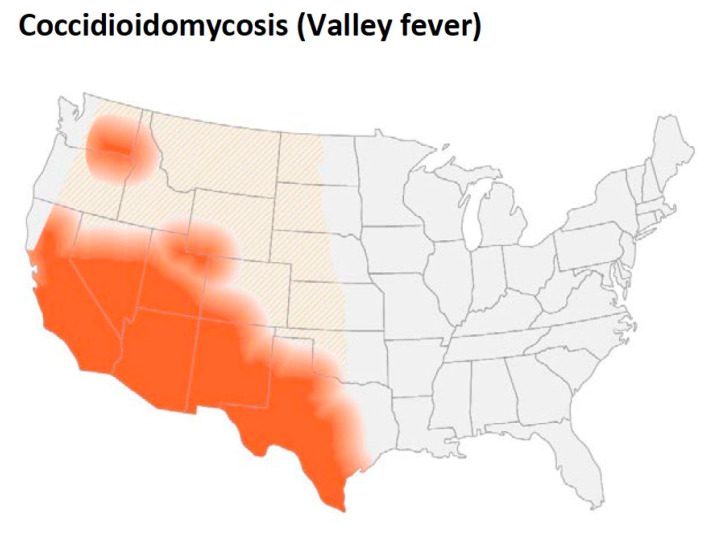
Updated map from the CDC estimating the distribution of *Coccidioides* spp. in the United States. This figure is from the Centers for Disease Control and Prevention (https://www.cdc.gov/fungal/diseases/coccidioidomycosis/causes.html) (accessed on 10 August 2022).

As Figure 4 shows, in addition to California (especially the San Joaquin Valley), southern Arizona, southern New Mexico as well as west Texas are endemic regions. Smith pointed out that the organism was also endemic in areas in Argentina, Venezuela, and northern Mexico [26]. He also reported more detailed geographic data about distribution of the disease in California. The incidence was maximal in the southern San Joaquin Valley, and some cases were seen in Monterey and San Luis Obispo counties. In fact, coccidioidomycosis is the most common fungal infection in stranded sea mammals in that area [28]. In addition to these areas, small foci have been identified in north-eastern Utah and eastern Washington [29,30,31,32].

The ecological factors associated with these geographic limitations have been investigated by many groups. It was first noted that the endemic region was in the Lower Sonoran Life Zone [25]. This region corresponds to the hot deserts of the south-western United States and north-west Mexico (the Mojave, Sonoran, and Chihuahua deserts) where the soil is hot, dry, and alkaline. The vegetation includes the creosote bush (*Larrea tridentata*) and the elevation of this region is between 100 ft to 3500–4000 ft. Total annual precipitation averages 10 inches or less. However, recovery of *Coccidioides* spp. from the soil by standard fungal culture was generally very poor (0–15% of soil samples) and inconsistent from samples that were near each other. Recovery of *Coccidioides* spp. seemed to be more common close to animal burrows [33]. In addition, evaluation of desert rodents showed that some of them were infected both in Arizona [34] and California [25].

The incidence of clinical disease varies substantially from year to year, which is thought to reflect the amount of fungus in the soil. Weather is clearly an important factor; relatively wet winters followed by months with little or no rain are associated with high incidence of disease [35,36,37]. A longitudinal study of a plot of soil also found that there were large differences in the recovery of *Coccidioides* spp. from year to year [38]. Recovery of *Coccidioides* spp. was higher in years when high levels of sodium, calcium, chlorides, and sulfates were found in the topsoil. *Coccidioides* spp. have not been reported from soil that is currently being farmed, [39,40], although farmworkers aren reported to be among the occupations at higher risk for coccidioidomycosis [41].

A small number of clinical infections have been identified in the south-eastern region of Washington and *C. immitis* has been detected in the soil by PCR and recovered by culture [30]. A systematic study of of soil in this area has been done [42]. The authors found that the organism was present in several locations within an 46,000 m^2^ area and persisted there for years. Soil with high levels of boron, calcium, magnesium, sodium, and silica was more likely to contain *C. immitis* and, in laboratory experiments, serilized soil contained enough nutrients to support the growth of the organism. They reported no association of colonization with animal burrows, but reanalysis of the data while accounting for likelihood of detection showed that samples from animal burrows were more likely to be positive than those away from burrows [43].

Another potential factor that is not usually considered is the presence of organisms in the soil that inhibit the growth of *Coccidioides* spp. A study in Bakersfield California found that some bacteria isolated from local soil inhibited the growth of *C. immitis* in vitro [44]. The organisms with this activity were determined to be closely related to *Bacillus subtilis* or *Streptomyces candidus*. Presumably, these organisms in the soil could influence the amount of *C. immitis* present and/or the ability to recover the fungus by culture. Other soil fungi probably play a role too.

Barker has recently reported that *C. posadasii* is very difficult to recover from soil by culture in vitro [24]. Even when *C. posadasii* is spiked into sterile soil the colony recovery rate is only 0.2%. Therefore, in her hands, soil culture is not a useful tool for estimating the amount of *C*. *posadasii*. They also used a mouse inoculation technique to recover organisms. BALB/c mice were inoculated intraperitoneally with a saline extract of 5 g of soil and monitored for mortality or symptoms of infection and those that appeared to be infected were examined by histology and culture to prove that *Coccidioides* spp. was the cause of their illness. Using this technique, 11/124 (9%) of the soil samples from the Tucson Arizona area contained *C. posadasii*., so this is a much more sensitive technique than soil culture. Similar studies have not been done with *C. immitis*, which can be cultured from soil [25].

The use of PCR to detect fungal DNA in soil has been a major step forward since it is a very sensitive test for the detection of the organism. Several tests are available that use a variety of targets including the internal transcribed spacer 2 region, a transposon, and other genes [45,46,47,48,49]. The sensitivity of the tests is largely dependent on the number of repeats of the gene target in the genome. In fact, the method of choice for detection of *Coccidioides* spp. in environmental samples is a qPCR test based on a multi-copy transposon unique to the genus [50]. Detection of *Coccidioides* spp. in soil is routine, but detection from air is problematic [51].

More recent ecological models have used much more sophisticated techniques [52]. Several sites around Bakersfield California are known to contain *C. immitis* DNA in the soil. A Landsat image of Kern County was used to evaluate the vegetation and soil temperature and a *Coccidioides*-likelihood of detection score was calculated. In 25 sites there was a reasonably good correlation (75%) between a high likelihood score and detection of the organism in soil by PCR. The type of vegetation might be a proxy for the type of soil and climate conditions, but no detailed plant diversity data were presented to support that idea.

Another model used the maximum entropy algorithm to combine bio-climactic and geographic variables to estimate the likelihood of *Coccidioides* spp. colonization [53]. The predicted likelihood was compared to evidence of soil colonization by PCR and the correlation was excellent (AUC > 0.944). Predictions were also made for the geographic distribution of desert rodents: *Neotoma lepida* (the desert woodrat) was the most closely associated rodent. The model predicts an increase in the geographic distribution of *Coccidioides* spp. to the north and east as the environment gets warmer, and drier as is expected in the future.

To make predictions on a nation-wide scale, a model was developed using data from a variety of soil, temperature, and rainfall databases that spanned the entire U.S. and then building a fuzzy machine learning model [54]. The authors found that the predicted suitability for growth of *Coccidioides* spp. correlated fairly well with the areas of highest incidence of disease. Comparison to PCR studies of soil also showed reasonably good correlation. This model identified the most well-known endemic areas in the south-west, but it also predicted the area in eastern Washington State where there is an endemic focus, further validating the usefulness of this model.

Desert animals are clearly infected with *Coccidioides* spp. and evaluating evidence of infection in them is a very useful technique to evaluate the extent of endemicity [24,33,55]. It has been proposed that persistence of the organism within granuloma in desert rodents and subsequent growth in the dead animal is a critical component of persistence in the environment [55,56]. One modeling study has found that regions to be colonized with *Coccidioides* spp. were also predicted to be colonized with the desert woodrat [53]. Another animal that has been associated with infection in South America is the armadillo, where people who extracate this animal from burrows have become infected [57] and *Coccidioides* spp. has been cultured from a few animals [58]. However, it is not clear how large a role animal infection plays in the persistence of the fungus in desert soils.

The small animal hypothesis is bolstered by the observation that gene families coding for enzymes that can digest animal proteins are expanded in *Coccidioides* spp. genomes while genes for digestion of plants are lost (see Section 6) [59]. In addition, this hypothesis is attractive because it suggests a potential reason why the ability to differentiate into spherules and infect animals might be evolutionarily beneficial. The small animal hypothesis is certainly intriguing but does not preclude a role for growth outside animals. For example, there are reports that sterilized soil supports the growth of *Coccidioides* spp. [42,60] but hyphal growth in soil supported by rodent carcasses or other substrates could increase spore production and aid in dispersal as well as transmission to naïve hosts.

It is clear that the desert regions of the southwest U.S. and northern Mexico are highly endemic, but it is also clear that the organism is found in the soil of other semi-arid regions. In addition, the endemic region seems to be expanding. More studies of *Coccidioides* spp. in soil, infections in animals and epidemiologic studies are needed to better define the endemic areas and the risk for infection in those areas.

## 4. Culture, Detection and Morphology of Mycelia and Spherules

*Coccidioides* spp. grow well on many bacteriological media (including blood agar) and standard fungal culture media such as Sabouraud’s dextrose agar and colonies usually appear within several days. As with other dimorphic fungi, the fungus grows as a mold at room temperature to 35 °C at a pH of 5–8. The initial growth consists of hyphae without internal arthroconidia. If pigmentation occurs, it is usually on the underside of the colony [61]. Arthroconidia usually develop within mycelia after one to two weeks but can take longer. *Coccidioides* spp. arthroconidia are highly infectious so all manipulations of the organism or with suspected isolates should be done in a BSL 3 biosafety hood. *Coccidioides* spp. can be identified in the appropriate clinical setting after the development of arthroconidia, but nucleic acid tests have been developed to identify the organism and, in some cases, to distinguish *C. immitis* from *C. posadasii*. The first molecular test was a DNA probe for identifying a colony as *Coccidioides* spp. (http://www.hologic.ca/products/clinical-diagnostics-and-blood-screening/assays-and-tests/accuprobe-culture-identification) (accessed 10 August 2022). There is one FDA-approved test for identification of *C. immitis* and *C. posadasii* DNA that distinguishes between the two species that has also been approved for direct testing of clinical specimens without culture (https://genestatdiagnostics.com/coccidioidomycosis-valley-fever/) (accessed 10 August 2022). Several other tests are also available for detection of *Coccidioides* DNA in clinical specimens [45,46,47,48], one of which also distinguishes between the two species [49].

When the organism is grown in liquid media (usually in the research laboratory) the hyphal form is obtained [62]. The most typical culture conditions are glucose yeast extract media at a temperature of 25–30 °C in the air with shaking. The maximal number of arthroconidia, develop after 4–6 weeks of culture when grown on solid medium.

### 4.1. Mycelia and Arthroconidia

**Figure 5:**
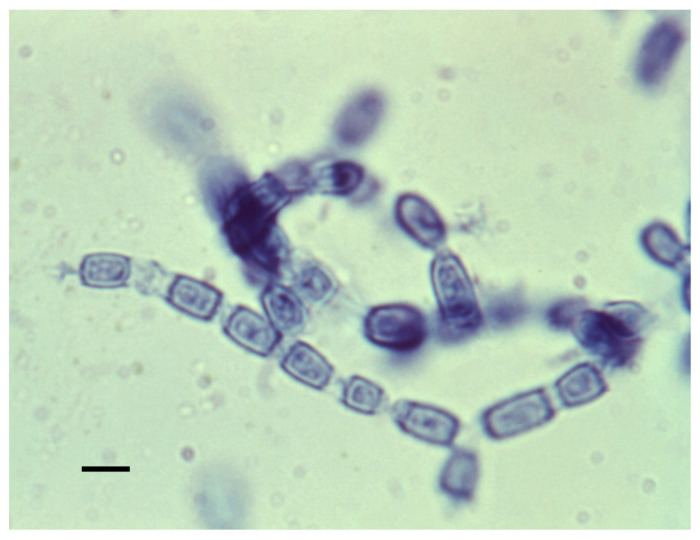
Arthroconidia within mycelia. Every alternate cell has degenerated. The bar is 5 μM. The figure is from the Centers for Disease Control and Prevention. (https://phil.cdc.gov/Details.aspx?pid=15780) (accessed on 10 August 2022).

The mycelial form is shown in Figure 5. Mycelia enlarge by apical growth followed by the development of septa. Once the septal spores close, every other cell degenerates, and the organism forms arthroconidia within a thin outer wall that is easily ruptured by the wind, resulting in their release. The arthroconidia contain two to five nuclei while the nuclei in the degenerating cell autolyze [63]. The arthroconidia can either develop into mycelia (in the environment) or differentiate into spherules (in mammalian hosts).

**Figure 6:**
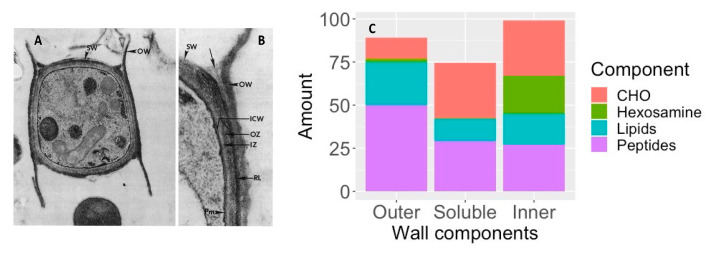
Components of arthroconidia cell wall. A and B: Thin sections of conidia of *C. immitis* showing differentiation of wall layers. OW, outer wall layer; SW, septal walls of conidium; ICW, newly formed inner conidial wall composed of the outer zone (OZ) and more homogeneous inner zone (IZ); RL, rodlet layer; Pm, plasmalemma. Arrow locates soluble conidial wall fraction (SCWF) trapped between OW and RL which is released during the cell-shearing process. (**A**), magnification ×12,000; (**B**), magnification ×27,000. The figure is from [64] and is reproduced with permission. (**C**), composition of inner and outer cell wall and SCWF. The data for this figure is from [64].

Arthroconidia have an inner and outer wall with an inner zone and a rodlet layer between the two as shown in Figure 6A. The outer wall is a remnant of the outer sleeve of the mycelium. To determine the chemical composition, the outer wall and rodlet layer were sheared off together and separated from the soluble inner zone and the intact, viable organism [64]. Figure 6C shows that the outer cell wall is rich in peptides and lipids, with relatively little carbohydrate, which probably contributes to its hydrophobic characteristics and its ability to become airborne and dispersed in the environment, including the airways of the mammalian host [64]. The large amount of hexosamine in the inner wall is primarily contained in chitin. In addition, the unusual sugar 3-O-methylmannose was found to account for 12% of the carbohydrate in the inner wall. This sugar has not been found in any other fungi except *U. reesii* [65,66]. Once arthroconidia are inhaled they begin to enlarge and their outer wall ruptures within 24 h. The soluble conidial wall fraction (SCWF) consists primarily of carbohydrates and peptides, with little lipid or hexosamine.

### 4.2. Spherules

**Figure 7:**
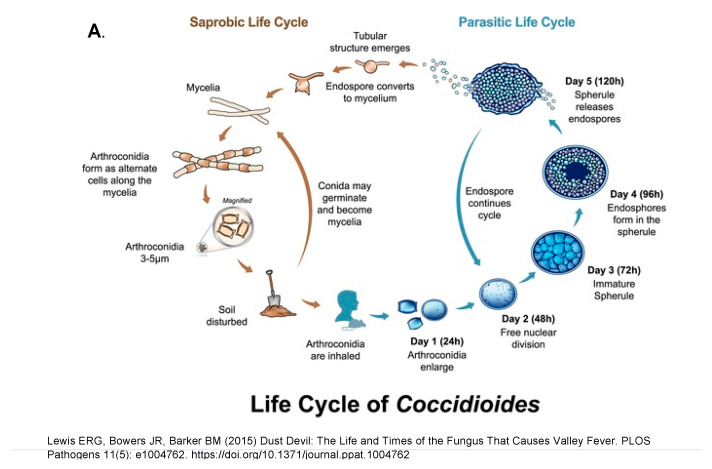

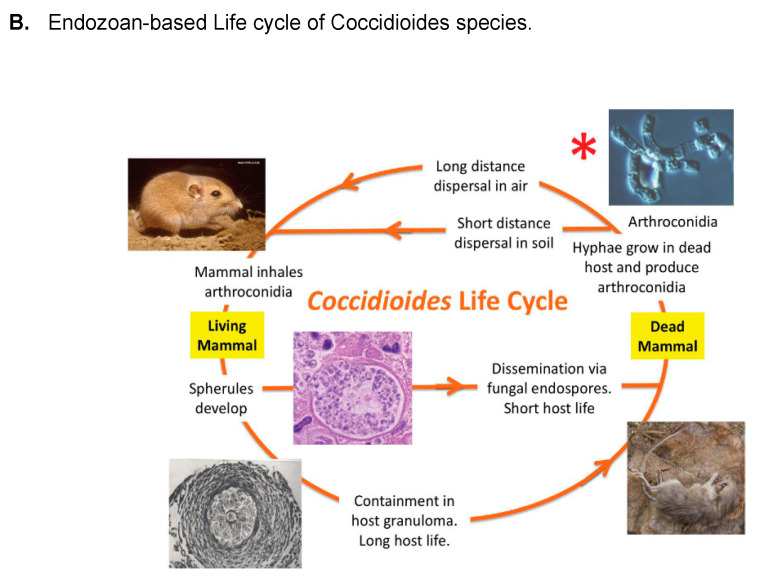
The saprobic (mycelia and arthroconidia) and parasitic (spherule and endospore) life cycle. The figure is from [65]. (**A**), mycelia growing in the soil and differentiating into spherules in animals. (**B**), Endozoan-based life cycle of *Coccidioides* species. Beginning at the asterisk (*), arthroconidia travel from the hyphae that produced them short distances among small mammals in burrows, or longer distances above ground, to infect other animals. The arthroconidia convert to spherules and are either controlled by the immune reaction or develop endospores, which disseminate to produce grave disease. The infected animal dies, either from disseminated coccidioidomycosis or from other causes and, in either case, living *Coccidioides* present in the animal, now freed from the host immune system and living at lower temperatures, convert to hyphae. The hyphae grow through the dead animal and then produce abundant arthroconidia, which initiate a new cycle of life for the fungus. The figure is from [55] and is reproduced with permission.

Differentiation into a spherule begins with the arthroconidium swelling into a round cell that then enlarges isotropically and divides internally to eventually form endospores. The round cell initially contains one nucleus, and the number of nuclei increases as internal division occurs leading to endospores with one nucleus each. A large spherule can contain hundreds of endospores. The spherule to endospore cycle begins when the spherule ruptures and releases endospores. Figure 7A represents the life cycle from soil to humans, while Figure 7B emphasizes the role of small mamals.

Spherule initials begin to divide by forming one cleavage plane, followed by another at 90° to the first, a third at 90° to the second, and so forth. The final stage of spherule development is the release of endospores by mature spherules. One group has reported that endospores are released in relatively large packets covered by fibrillar material [67]. The authors hypothesized that the packets of endospores might be more difficult for white blood cells to phagocytose than single endospores. However, only a few studies of endospore phagocytosis have been reported. In general, endospores are a stage of the organism that has not been well studied.

Arthroconidia differentiate into spherules very quickly in vivo. Animal studies show that the conversion happens in the lung, in the muscle, or in the peritoneum within 24–48 h of inoculation. One clever way of obtaining spherules in vivo is to implant a chamber covered by a dialysis membrane subcutaneously into mice, wait until the chamber spontaneously fills with extravascular fluid and then inject arthroconidia [68]. The chambers contain no mouse cells. Over the next ten days the organism grew as spherules (70%) and hyphae (30%). The addition of bronchial alveolar cells had no effect on the development of spherules. Removal of the chamber and culture at 37 °C in the air led to the development of hyphae but when cultured at 37 °C in 5% or 20% CO_2_ a substantial number of spherules were obtained [68]. Using buffers to obtain the same pH as was obtained in a CO_2_ environment did not stimulate spherule growth. The authors concluded that an elevated pCO_2_ played a major role in spherule growth.

There are two general methods for growing spherules in vitro. One is the Converse medium, which is a synthetic medium of salts with glucose as the nutrient [69]. Ammonium acetate is the primary salt, with NaCl, NaHCO_3_ as well as potassium phosphate, calcium chloride and zinc sulfate present in lower concentrations. The pH is adjusted to 6.5. Usually the detergent Tamnol-N is added which is thought to enhance the release of endospores [70]. A solid medium can be made by adding agar to the medium. The growth conditions are critical: a temperature of 35–40 °C and 5–15% CO_2_ with shaking of liquid cultures is required. Mead and Barker have recently provided a detailed protocol for growing spherules [62]. They recommend adding fresh medium if the spherules are maintained in culture for more than five days to facilitate endospore release. In some *C. immitis* strains spherules develop within two days [71] although most studies have used spherules after three to four days in culture [15,72]. The pace of development of immature spherules to endospore release in vitro differs between experiments with values from four days to more than eight days which may be influenced by strain differences. After the release of endospores, the second generation of spherules becomes asynchronous quickly.

One group studied the effects of amino acids, vitamins and substituting other carbon sources on the conversion from arthroconidia to spherules [73]. Phenylalanine, tyrosine, tryptophan, dihydroxyphenylalanine (DOPA) and pyrocatechol, singly or in combination, increased the rate of endosporulation. They argued that phenylalanine conversion to tyrosine, conversion of tyrosine to DOPA, followed by conversion of DOPA to melanin might be important for the formation of mature spherules.

Another study investigated the effect of human sex hormones on spherule growth since males and pregnant women in the third trimester are more likely to develop disseminated disease than the general population [74]. Spherules, grown in Converse medium, were exposed to 17β-estradiol, 17α-estradiol, progesterone, or testosterone. All these hormones, except for 17α-estradiol, (which is an inactive stereoisomer) stimulated the growth of spherules at physiologic concentrations of hormones. Glucocorticoids did not stimulate spherule growth. 17β-estradiol also stimulated the growth of the organism in the mycelial phase. In a follow-up study, both a saturable high-affinity and low affinity-binding activity for progestin, estrogen, and androgen hormones were detected in spherule cytosol [75].

RPMI is a medium that is designed for the growth of mammalian cells, but it also has been shown to support the growth of spherules. The initial study used RPMI with 10% fetal calf serum and 0.8 mg/mL N-Tamnol in 5% CO_2_ at 35 °C with shaking [76]. By 48 h, about 10% of organisms were in the spherule form. Hyphae were removed by filtration and spherules were collected by centrifugation. This process was repeated and after four passages essentially all the organisms were spherules. The N-Tamnol was important for rapid and complete conversion to spherules but once conversion occurred it could be eliminated. A recent study found that spherules grew larger and to a higher density in RPMI than in Converse medium and that growth in 1% O_2_ (with CO_2_) was preferable to air (with CO_2_) [72]. Other experiments have found that 10% fetal bovine serum or 0.1% Survanta in RPMI both support the growth of spherules [77]. There is one study comparing spherules of several strains of *C. immitis* and *C. posadasii* by scanning electron microscopy [72]. There appeared to be a difference in the surface of the spherules, with *C. posadasii* Silveira having a more wrinkled surface than the other strains. Characterizing the differences between hyphae and spherules has been a major area of investigation (see Section 8.1, Section 8.2 and Section 8.3).

Some methods for inducing the transformation of arthroconidia into spherules are well established but the detailed biological mechanisms of the differentiation are still a mystery. Some studies show that protein profiles of spherules induced by growth in Converse medium are different than spherules grown in RPMI (with different additives) or to spherules in vivo [78]. These observations indicate the limitation of studies of spherules grown in vitro. In addition, there has been little investigation of the localization of proteins within the fungus during the transformation. Finally, there has been almost no investigation of endospore to spherule differentiation.

## 5. Antigens

### 5.1. Dermal Hypersensitivity or Skin Test Antigens

The motivation for the first study of *Coccidioides* spp. antigens was to develop reagents for skin testing and serologic testing. Early in the study of the disease it was appreciated that delayed-type hypersensitivity (DTH) was important for the pathophysiology and for the diagnosis of the infection [79,80]. The model for detection of DTH was the tuberculin skin test.

The initial skin test antigens were obtained from a combination of the supernatant of hyphal culture and the toluene extract of the cells [26]. This preparation, known as coccidioidin, was remarkably stable to heat, and antigenic activity was found in high molecular weight fractions containing both protein and carbohydrate, including 3-O-methyl mannose [81]. Skin testing with coccidioidin was very sensitive in infected animals and people with a clinical history of the illness, and was specific for coccidioidomycosis [79].

Antigens derived from spherules were investigated once techniques for the culture of spherules in vitro became widely available. Spherulin has been widely used as a skin test reagent and it elicits a positive skin test more frequently than coccidioidin in both experimentally immunized animals and immune persons [82,83]. The level of false-positive reactions with both spherulin and coccidioidin is very low. There were at least two approaches to the preparation of spherulin. One was to simply use the Converse medium culture filtrate [84] and another was to extract material from spherule cell walls by mechanical disruption, followed by extraction with NaOH [85] or toluene [86] and to add the extracted material to the culture filtrate. These reagents were commercially produced until the 1990s when they became unavailable. Spherulin containing 0.4% phenol as a preservative has recently become available as a commercial skin test (Spherusol) [87,88]. Skin testing can be used both as an epidemiologic and a clinical diagnostic tool, although its diagnostic usefulness in a highly endemic area is limited because so many people are skin test positive from undiagnosed prior infections. Extrapolating from that, we can assume that many infections are asymptomatic or so mild they are not remembered.

### 5.2. Characterization and Purification of Antigens from Coccidioidin

**Figure 8:**
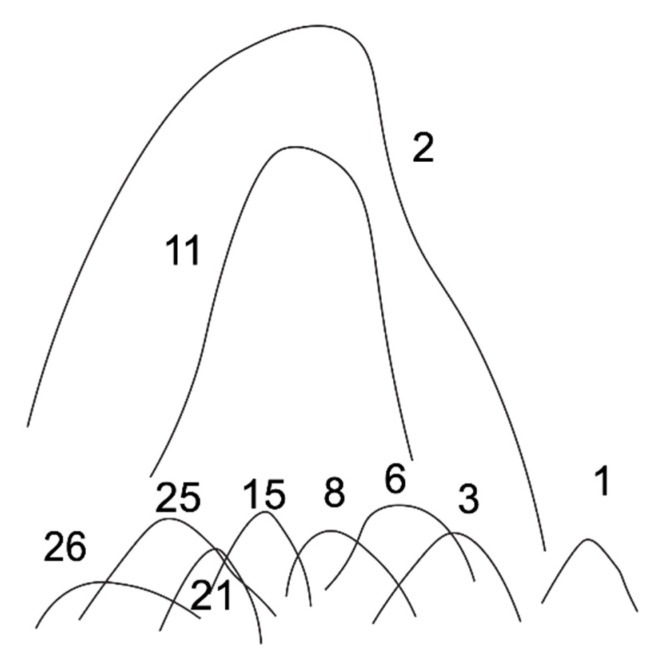
Two-dimensional immuno-electrophoresis precipitin lines. Drawing of some typical precipitant lines in two-dimensional immuno-electrophoresis of coccidioidin with anti-coccidioidin antisera. The antigens are numbered as described [89].

In an attempt to characterize coccidioidin, Huppert and Sun did experiments with two-dimensional immuno-electrophoresis with anti-coccidioidin antisera raised in burros [89]. The formation of antigen–antibody complexes led to precipitation in the gel. A typical pattern of precipitin lines is shown in Figure 8.

There are a total of 26 antigens detected by this method and a smaller number of antigens were appreciated using this technique with spherule extracts. The most abundant antigen is antigen 2 (Ag 2). It is certain that there are many more proteins in coccidioidin than are identified in this study, including an antigenic protein designated the *Coccidioides*-specific antigen (CSA) [90]. In fact, modern proteomic techniques have identified more than 800 proteins in coccidioidin [91]. Ag 2, 11 and CSA were isolated and characterized [92,93,94]. Furthermore, subsequent studies resulted in DNA cloning, the expression of recombinant protein, and the evaluation of the Ag 2 and CSA as vaccines [95,96,97].

### 5.3. Complement Fixation Antigen

The complement fixation (CF) antibody test is a sensitive, specific, and prognostic test that has long been recognized as a major serologic tool for the diagnosis and management of coccidioidomycosis [98]. In one early study, Pappagianis and his colleagues reported that a partially purified protein found in spherule culture filtrates containing the CF antigen also contained chitinase activity [99]. The protein bound to chitin and a single band in SDS-PAGE had chitinase enzyme activity. In immunodiffusion assays with the crude CF antigen, the partially purified protein formed a line of identity, confirming that it was also antigenic.

The amino acid sequence of this protein was determined [100] and used to identify the coding DNA [95,101,102]. Two chitinase genes were identified and Chitinase 1 (*CTS1*) was thought to correspond to the major protein in the CF antigen. In a subsequent study, deletion of the *CTS1* gene did not reduce the virulence of the organism or the progression to the release of endospores in vitro, suggesting that the enzyme was not required for normal growth or pathogenicity [101]. However, *Coccidioides* spp. have at least eight different chitinase genes [102], thus redundant gene functions may complement the deleted enzyme. In a more recent study, the *CTS1* gene was expressed in the closely related species *U. reesii,* and the recombinant protein was purified [103]. The purified recombinant protein had chitinase activity and the protein formed a line of identity in immunodiffusion CF antigen. Evaluation of patient sera with or without positive CF titers showed a clear difference in titer to Cts1 protein and a positive correlation between ELISA with the recombinant Cts1 protein and the CF titer obtained with crude antigen. Based on these studies, it seems clear that Cts1 is a major component of the CF antigen, although it is not clear whether other antigens also contribute. Experiments with immuno-depletion would be useful to address that issue.

### 5.4. The Precipitin Antigen

The precipitin antibody test detects IgM antibody. Historically, it was detected by visualizing antigen–antibody aggregates in liquid. Since this antigen detects IgM antibody to *Coccidioides* spp., it is useful for the diagnosis of the disease in its early stage [98]. It was recognized that antigenic activity could be enriched from coccidioidin by concanavalin A binding, indicating that it was a carbohydrate or a glycoprotein [104]. IgM antibody from patient sera recognized two partially purified proteins in an ELISA assay [105]. The antigenic activity was not destroyed by pronase but was inactivated by periodate treatment, indicating that carbohydrates (including 3-O-methylmannose) were required for antigenic activity. The purified protein contained β-1,3-glucanase activity which was found in the spherule cell wall and cytoplasmic vesicles [106]. The genes coding for two β-1,3-glucanase proteins were cloned and recombinant proteins were expressed [107]. The *BGL2* transcript was expressed at relatively high levels in spherules at 36 and 132 h of culture in vitro, which are periods of isotropic growth.

The *BGL2* gene has recently been expressed in *U. reesii* and tested for immunoreactivity with patient serum [66]. The Bgl2 protein was glycosylated with 3-O-methylmannose. In IgM ELISA assays, the recombinant protein was detected by 79% of patient sera, which is similar to the results obtained with the MiraVista Diagnostic IgM antigen. In patients with illness lasting <3 months, 83% of the patient sera were positive, although there was a 17% false-positive rate. The recombinant protein was more sensitive than commercially available proprietary antigens for IgM ELISA’s although the recombinant protein was somewhat less specific.

### 5.5. Purification of Antigens Based on the Huppert System

Experiments that attempt to purify and characterize antigens based on Huppert’s two-dimensional electrophoresis are difficult to follow because somewhat different methods were used to obtain the crude starting material and those were given different names (alkali-soluble, water soluble cell wall extract or C-ACWS, compared to soluble conidial wall fraction or SCWF and toluene spherule lysate or TSL) and the functional characterization of the partially purified proteins also varied. For these reasons, the terms used to designate these antigens proliferated.

Ag 2 was the most common antigen in coccidioidin and a monoclonal antibody to the protein was used to detect the protein in a recombinant DNA expression library [95]. The gene was sequenced, and the predicted protein was found to contain multiple threonine proline repeats. The recombinant protein elicited a positive footpad swelling response in immune mice, suggesting that this protein was an important antigen for T-cell mediated immunity in mice.

Another group of investigators also investigated Ag 2. Using TSL as starting material, they found that the crude product was highly glycosylated (about 35 times more sugar than protein) and contained Ag 2 as well as several other antigens in the two-dimensional immuno-electrophoresis system. Immune patient sera reacted with a wide variety of molecular weight bands in immunoblots, which is also consistent with glycosylation and/or multiple proteins in the crude lysate [86].

The product was deglycosylated and the protein was partially purified. This protein had an apparent molecular weight of 33 kD on SDS-PAGE and was proline- and threonine-rich. It reacted with immune patient sera [93]. Using an antibody raised against the partially purified protein, the cDNA was cloned from an expression library [96]. The recombinant protein (named the proline-rich antigen or PRA) elicited a lymphocyte proliferation response in immune mice and was a protective vaccine in a variety of experiments [108,109]. The predicted protein was the same as the Ag 2 protein that had been cloned and expressed by Cox and colleagues but was given a different name. Because of confusion about this redundant nomenclature, the protein was designated Ag2/PRA in later studies.

The gene coding for antigen CSA was cloned, and a recombinant protein was expressed [97]. Both CSA and Ag 11 were found to have proteolytic activity [110,111,112]. Immune human serum did not bind to Ag 11 in ELISA, but it did bind to CSA. Ag 11 was found to be a chymotrypsin-like serine proteinase [113]. It was found in the chitin-rich areas of the fungal cell wall and proposed to play a role in digesting wall proteins and influencing cell wall remodeling.

A recent study of human immune serum identified at least 46 antigenic proteins and glycoproteins in the spherule cell wall [114]. The proteins were separated by 2D-electrphoesis and immunoblotted with human immune serum. A number of antigens that had been previously identified were seen (chitinase 1, SOWgp, Ag2/PRA) but many novel antigens were also identified. Some of these have been incorporated into a multivalent recombinant vaccine that is currently being tested [102].

### 5.6. Antigens for In Vitro Measures of T-Cell Mediated Immunity

One of the first methods of evaluating T-cell mediated immunity in vitro was the lymphocyte proliferation assay. It was reported in 1974 that both spherulin and mycelial culture filtrate stimulated lymphocyte proliferation in immune but not non-immune people [115]. The stimulatory activity was not dialyzable and was maximal at relatively high concentrations.

A different approach was taken to identify an antigen that stimulated antigen-specific T-cell proliferation in mice. SCWF contains antigens that are recognized by antisera raised to coccidioidin [90]. This material also stimulated T-cell proliferative responses in murine T lymph node cells from mice immunized with a live, attenuated mutant of *C. immitis*. A T-cell line was made from spherule immunized mouse lymph node cells by repeated stimulation with SCWF. This T-cell line was found to preferentially respond to a 43–66 kDa fraction in an H-2 restricted fashion [90]. (H-2 restriction is consistent with an antigen-specific T-cell response.) The fungal gene coding for the antigen was cloned and expressed as a recombinant protein and found to be homologous to 4-hydroxyphenylpyruvate hydrogenase [116]. This gene is highly conserved in many species, including mammals. It has not been further investigated as a possible vaccine.

The purification and analysis of these proteins from crude extracts was a difficult task, especially since the methods at the time were much less sophisticated than those that are currently available. It is ironic that we still use crude antigens for diagnostic tests although we recognize that current diagnostic tests are suboptimal. Many novel diagnostic strategies have been proposed and may provide valuable new approaches, but a more extensive evaluation of the recombinant proteins that are already available for antibody and T-cell testing might be a significant step forward.

## 6. Genome Sequences

Sequencing the genome of *Coccidioides* spp. was an enormous breakthrough in our understanding of the organism and much of our current knowledge stems from that data. Before the whole genome sequence was available, defining a gene required a method to recognize it, cloning the cDNA and sequencing that cDNA. This one-by-one approach would have taken many lifetimes to identify and characterize a substantial set of important genes. Now the genomes of a fairly large number of *Coccidioides* isolates of both species have been sequenced and the majority of genes have probably been correctly predicted. However, it is almost certain that some genes have been mis-identified by the annotation process, or wrongly predicted, and many transcriptional variants of genes have not been appreciated.

The initial analyses were done with *C. immitis* RS and *C. posadasii* C735, two strains that had been used for laboratory experiments as well as the closely related, but non-pathogenic organism, *U. reesii* [59]. The assembled genomes for *C. immitis* RS and *C. posadasii* C735 are available from NCBI–ASM14933v2; *C. posadasii* C735–JCVI- cpa1–1.0. The depth of sequencing was 8–14-fold, which is lower than subsequent studies. (Depth of sequencing refers to the number of times DNA is sequenced). The genome size was nearly identical in *C. immitis* and *C. posadasii* (29 and 27 Mb) but the number of predicted genes was substantially different: 10,355 genes were predicted in *C. immitis* compared to 7229 in *C. posadasii*. This variation most probably results from the use of different annotation methodologies. There was a mean of 2.3 introns per gene and the introns were about 100 bp long. Twelve percent of *C. posadasii* DNA and seventeen percent of *C. immitis* DNA was repetitive. Only 4% of *U. reesii* DNA was repetitive.

Seven overlapping continuous DNA segments (contigs) were predicted in *C. immitis* compared to previous estimates of four chromosomes obtained by electrophoresis [117]. However, some of the predicted contigs were very similar in length and they might not be resolved by electrophoresis. Subsequent more-complete studies have shown that *C. posadasii* Silvera has six chromosomes [118,119]. The size of gene families in Onygenales and Eurotiales taxa was compared [59]. Many genes involved in the catabolism of plant cell walls were not found in Onygenales. The only two gene families that were expanded were APH phosphotransferase, coding for protein kinase activity, and a subtilisin N domain-encoding family.

**Figure 9:**
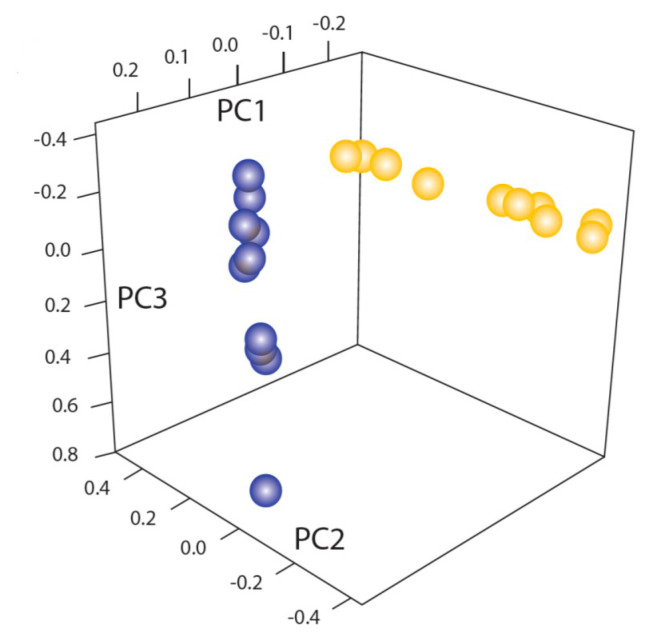
Principal component analysis of the genomic sequence of *C. immitis* and *C. posadasii* strains. The blue points represent DNA sequence of *C. immitis* strains; the yellow points represent DNA sequence of *C. posadasii* strains. The figure is from [120].

Another study of ten strains of *C. immitis* and ten of strains *C. posadasii* isolates was done [120]. The best estimate of the number of contigs was six. A principal component analysis demonstrated the consistent genetic difference between the isolates of the two species, confirming the divergence between the two species (Figure 9).

One major finding of this paper was that there was some recombination between *C. immitis* and *C. posadasii* with the primary direction of gene flow being from *C. posadasii* to *C. immitis*. Recombination is discussed in more detail in the next section. The amount of repetitive content varied from 8 to 21% and gypsy transposons were the most common type. There was some evidence of repeat-induced mutation to control repetitive DNA, especially affecting CpG and CpC dinucleotides.

Another *C. immitis* strain recovered from the soil in Washington has recently been sequenced and annotated [121]. The results are available at NCBI (GCA_004115165.2). The analysis of this sequenced genome predicted 7815 protein-encoding genes. Almost all the proteins predicted by this analysis were very similar to the *C. immitis* RS annotation (orthologous with a *p*-value < 10^−15^). However, the total number of predicted proteins in the analysis is much closer to the numbers of predicted proteins for the other isolates of *Coccidioides* spp.

There are currently five assemblies of different *C. immitis* strains and ten *C. posadasii* genome assemblies are available at NCBI. The same data is available at FungiDB (https://fungidb.org/fungidb/app/) (accessed on 10 August 2022). Some of the gene data is also available from UniProt (https://www.uniprot.org/) (accessed on 10 August 2022) but the gene designations are different.

A chromosomal-level sequence of *C. posadasii* Silvera has also recently been obtained [118,119]. This sequencing project relied heavily on Single Molecule Real-Time Sequencing to obtain the sequence of long reads as well as short-read sequencing to improve the quality of chromosomal sequence. Six chromosomes were predicted, as well as a mitochondrial chromosome. The sequence was similar to the *C. immitis* RS sequences, except for a region on contig 2 which appears to be an inversion. Very sophisticated software was used to predict gene models and 8500 genes were predicted. Secondary metabolites, such as polyketides, play a role in pathogenicity of other fungi and 23 gene clusters involving secondary metabolism were identified, which is similar to previous estimates [122]. This paper is clearly the most definitive description of the *C. posadasii* genome as of 2021 and a similar effort in a *C. immitis* strain would be very useful.

It might be useful to define a core set of genes that are shared between multiple isolates of *C. immitis* and *C. posadasii*, since it would seem likely that this set would include genes that are required for the growth of the organism and the differentiation into spherules. Orthologs shared between *C. immitis* RS, H538.4, WA_211 and *C. posadasii* Silvera and RMSCC 3488 have been identified. A total of 7950 genes are common to all these annotations (Supplemental Table 1 from bioRxiv 413906; doi: https://doi.org/10.1101/413906, accessed on 10 August 2022). Highly conserved *Coccidioides* spp. genes that are not present in non-pathogen Onygenales might contain some that play a role in pathogenesis. Genes that are found in the conserved *Coccidioides* spp. set but not in the non-pathogenic Onygenales are also found in Supplemental Table 1 in that manuscript.

### 6.1. Gene Annotation by Proteomics

Proteomics is a complementary approach to identifying predicted coding genes. The proteins from coccidioidin and spherulin produceded from *C. posadasii* Silveira have been examined [91]. A total of 837 proteins were identified, 88% of which were annotated in *C. posadasii* annotation and 9% were found in the *C. immitis* annotation (the remainder were annotated in other *C. posadasii* strains). Twenty percent of the proteins that were identified were classified as hypothetical proteins by DNA gene annotation, confirming that the predicted proteins were expressed. A total of 288 novel proteins were identified. Most of those were intragenic or novel exons indicating disparities between the predicted intron/exon structure and protein products. Hopefully, annotations from the improved *C. posadasii* Silveira described above will address some of these issues. Although only eight percent of the total number of predicted proteins were detected by proteomics, this is a similar fraction to the number of proteins identified by proteomics in *Aspergillus fumigatus* [123]. It is probable that many proteins were difficult to extract from the organism or were present in small amounts.

### 6.2. Transposable Elements

In addition to the analysis of repetitive DNA done in the genome sequencing studies, other studies of transposable elements (TE) in *Coccidioides* spp. have been done. One highly sensitive and specific PCR detection method is based on amplification of a transposon [50]. Another analysis found that LTR/Gypsy was the most common type, as had been previously reported [124]. Only 20–30% of Gypsy TEs contain ORFs with polyprotein domains, indicating that most have been extensively modified. In *C. immitis* RS, genes within 1 kB of one or more TEs were expressed at a lower level than those that were not close to a TE. This relationship was particularly evident with Gypsy and hAT TEs, but decreased expression was not observed in *C. posadasii*. In both species, genes involved in protein phosphorylation were enriched in the TE-associated group [124]. Expression of Gypsy, Copia, and TcMar transposons, determined by RNA-Seq, found that 27% of the transposons were expressed at a relatively low level. Most of these were preferentially expressed in spherules [16].

There is also proteomic support for expression of a partial transposon protein in *C. posadasii* [91]. These peptides mapped to multiple regions of the genome of both *C. posadasii* and *C. immitis* with homology to *gag* genes. The predicted TE protein contained gag, pol, reverse transcriptase, and integrase domains and was probably in the Gypsy family. Because of the presence of stop codons in the pol domain, it was thought that only the gag proteins were translated. The biological significance of these findings is unknown.

### 6.3. Resequencing and Phylogenomics

Much of the genomics work has focused on the phylogenomics of the organism. This type of analysis determines the genetic structure of the population, which is possible because of the limitation of gene flow between isolates that are geographically distant from one another. Many strains from many different regions as well as environment and clinical isolates have been sequenced to a much greater depth and aligned to assembled *C. posadasii* and *C. immitis* strains [125]. In one study of 68 strains there were about 13,000 high-quality, informative single nucleotide polymorphisms (SNPs), 40% of which separated the two species with the others being polymorphic in one or the other species.

Several clades within *C. posadasii* were found based on geography. Except for Guatemala, the other clades of *C. posadasii* diverged about 5000–8000 years ago. Analysis of 18 *C. immitis* strains showed a similar divergence over time, but there was less geographic clustering. Combined with other studies [22,32] at least two clusters are proposed, one embracing strains from Washington state and some strains from the the San Joaquin Valley of California, and the other embracing other strains from the San Joaquin Valley as well as those from Southern California and Mexico. A better understanding of *C. immitis* will require more sequenced isolates of *C. immitis*.

A follow up study about SNP-based phylogenomics has also been published [126]. This study also found that there were two clades within *C. posadasii*, one in Arizona, another in Texas, Mexico, and South America, and Venezuela and Guatemala.

Comparison of SNP data among strains can be used to detect evidence of natural selection in the form of positive selection, negative (or purifying) selection, selective sweeps of entire alleles, or hybridization and introgression [126]. In *Coccidioides*, initial evidence for hybrtidization and introgression, as well as selective sweeps, has been published [122,127], and more extensive analyses are encouraged.

If both strains and SNPs for those strains are available, a genome-wide association study for a given phenotype could be done, although the strains would need to be members of one, interbreeding population. The ability to associate alleles with variable traits is also dependent on balance of the variants in the population, and association has been detected in fungi from as few as 40 strains where the distribution of traits was balanced [128]. Unfortunately, only a few strains are currently available from a public repository.

### 6.4. Recombination and Sex

Although there are no published studies describing a sexual cycle in *Coccidioides* spp., there are many studies demonstrating that genetic recombination (cryptic sex) occurs. Taylor and colleagues used an arbitrary random primer PCR amplification to characterize DNA sequence polymorphisms [20]. They characterized 25 strains and found that there was recombination between the isolates, which is most consistent with cryptic sex. Subsequent studies with isolates from a more diverse group of isolates, including both *C. immitis* and *C. posadasii*, confirmed these findings [19,32,129,130]. When the genomic sequence of *C. immitis* and *C. posadasii* strains were determined it was found that a number of recombination events had occurred between *C. immitis* and *C. posadasii* [120]. High levels of recombination occurred in hot spots along the contigs.

A more detailed approach and extremely rigorous study has recently been reported [131]. The genomes of 51 *C. posadasii* strains and 27 *C. immitis* strains were examined for SNPs. The primary direction of introgression (transfer of genetic information) was from *C. posadasii* to *C. immitis* and most, but not all, of the introgressions segregated at low frequency. The vast majority (98% for both species) of introgressed haplotypes are small (less than 25 kB). Despite the preference for transfer from *C. posadasii* to *C. immitis*, more introgressed alleles were found in *C. posadasii* than *C. immitis*. The number of introgressed alleles varied across the genome. Some of the genes that were found in the introgressed regions were a variety of transporters and genes involved in metabolism. It is clear thatinterspecies genetic recombination occurs at a low rate; recombination within a species probably occurs at a higher frequency. 

Fungal mating is regulated by the mating type (*MAT*) locus. Most often, a given isolate expresses one of two alleles that code for transcription factors [132]. Mating type loci have been identified in multiple isolates of both *C. immitis* and *C. posadasii* and are found in equal frequency in both species [131,132,133,134]. One mating type is an HMG domain *MAT1-2,* and the presumed alternate type is a *MAT1-1* alpha box [32,135,136]. The balance of both mating types and the aforementioned recombination observed in natural isolates indicate that *Coccidioides* spp. have and employ the molecular machinery for sexual recombination. However, there are no published reports of visualization of mating and no recombination of haplotypes has been generated in vitro, although there is promising work in progress about identification of a sexual cycle.

## 7. Molecular Analysis of Spherules and Virulence-Associated Genes

### 7.1. Individual Genes

Since spherules are the pathogenic form of the organism and are unique to *Coccidioides* spp., understanding them and their development has always been a major focus of research. The studies of proteins and the associated genes based on hypotheses will be discussed first, followed by a discussion of more global approaches.

Seven chitin synthase genes have been identified and sequenced in *C. posadasii* [127]. One representative of each of the classes has been found. *CHS1* and *CHS4* mRNA are preferentially expressed in spherules, *CHS2*, *CHS3,* and *CHS6* are preferentially expressed in mycelia, and *CHS5* and *CHS7* are equally expressed in both phases. The *CHS5* gene has been deleted and the resulting mutant is avirulent.

The ornithine decarboxylase (ODC) gene has also been investigated [129]. The amount of mRNA expression did not vary as spherules developed and matured but the amount of enzyme activity peaked in the isotropic growth phase of spherules. Furthermore, a chemical inhibitor of ODC blocked development of spherules. The reason for this is not known and this line of research has not been pursued.

#### 7.1.1. Spherule Outer Wall Glycoprotein

Initial studies of a spherule outer wall found that an electron-dense extracellular matrix, referred to as hyphal outer wall layer, coated spherules [130]. Mature spherules were phagocytosed very poorly by human polymorphonuclear leukocytes, even in the presence of immune serum and the authors thought that this extracellular matrix might play a role in preventing phagocytosis [130].

**Figure 10:**
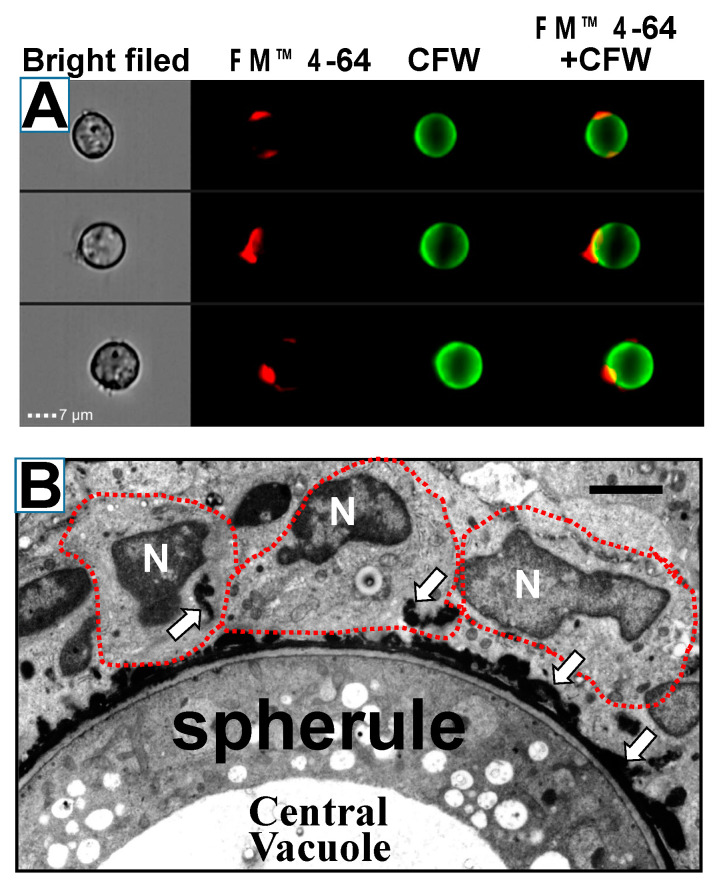
Images in triplicate of spherules (**A**) labeled with calcofluor white (CFW) and FM^™^4-64FX, a lipophilic dye (red) in vitro. SOW lipids are enriched in the inner leaflet of SOW. When the lipid is exposed as the SOW separates from the surface it binds to FM^™^4-64FX dye [135]. Electron microscopy image (**B**) of a histological section of *C. posadasii*-infected tissue from the mouse lungs. Tissue section was labeled with osmium tetroxide (OsO4) to enhance the visibility of lipids. Host phagocytes are outlined with red dash-dot lines and their nuclei are labeled (N). Lipid-rich SOW fragments are visible inside phagocytes (white arrows). The bar is 5 μM. Image courtesy of Dr. Garry Cole and Dr. Kalpathi R. Seshan.

Another group did microscopic studies of spherules grown in vitro which revealed a layer of material outside the spherule wall that enveloped the organism and was shed from the surface [137,138]. This material designated the spherule outer wall (SOW) contained both antigenic proteins and lipids (Figure 10) [111,138]. An octyl-glucopyranoside extract of this material, which contained a 66 kD protein (SOWgp), was recognized by immune patient sera and stimulated a robust lymphocyte proliferative response in immune mice and people [111].

The gene coding for SOWgp was cloned and expressed [139]. It consisted of four-six proline aspartate and cysteine-rich repeats (depending on the strain of *Coccidioides* spp.) with a glycophosphoinositol (GPI) anchor. The mRNA and protein were not detected in hyphae but were developed early in the process of spherule development. The gene was deleted by a targeted insertion and the resulting strain was tested for pathogenicity. The SOWgp deleted mutant was much less virulent than the parental strain (Experiment 1, Table 1).

#### 7.1.2. Urease and Ureidoglycolate Hydrolase

*Coccidioides* spp. produce a urease enzyme that produces ammonia and alkalinizes culture media [140,141]. Urease was found in intracellular vesicles and the central vacuole of spherules and was released when the spherules ruptured [142]. The urease gene was deleted, and the deletion mutant was tested for the ability to produce ammonia in culture and for pathogenicity in mice. As expected, no enzyme activity was seen. In addition, the urease deletion mutant was substantially less pathogenic than wildtype (Experiment 2, Table 1).

Another enzyme, ureidoglycolate hydrolase (*UGH*) catalyzes the hydrolysis of ureidoglycolate to yield glyoxylate and the release of CO_2_ and ammonia. Expression of UGH mRNA in spherules increased as spherules matured [136]. To address the role of this enzyme in the pathogenicity of *Coccidioides* spp., the *UGH* gene was deleted as well as deleting both *UGH* and *URE* and the pathogenicity of these deletion mutants was evaluated. Δ*ugh*/Δ*ure* double mutants made less ammonia than either mutant alone. The double mutant was much less pathogenic than the wildtype organism or either mutant (Experiment 2, Table 1). This data strongly suggests that the ability to produce ammonia and alkalinize the tissue around *Coccidioides* is a vital element of pathogenesis.

#### 7.1.3. Metalloprotease

A metalloenzyme protease (*Mep*1), which is preferentially expressed as spherules endosporulate and rupture has also been proposed to be a pathogenic factor [143]. This enzyme digests SOWgp from the surface of endospores. The *Mep*1 gene was deleted, and the deletion mutant was found to produce the same number of endosporulating spherules as controls, but significantly more SOWgp was associated with endospores in the mutant strain. The pathogenicity of Δ*mep*1 in non-immune mice was not reported but the mutant was less pathogenic than the wildtype organism in mice that had been immunized with SOWgp. The authors suggested that the anti-SOWgp antibody enhanced phagocytosis of endospores and that endospores were coated with more SOWgp in the absence of *Mep*1.

#### 7.1.4. Melanin

There is evidence that *Coccidioides* produces melanin in vitro and in vivo since arthroconidia, spherules, and endospores were stained with an anti-melanin monoclonal antibody [144]. Staining of the spherule by anti-melanin monoclonal antibody was seen in infected mouse tissues as well as in vitro. Melanin is a pathogenic factor in many other fungi and may also play a role in pathogenesis in *Coccidioides* spp.

## 8. Comparison of the Saprobic and the Parasitic Phases

### 8.1. Transcriptional Studies

An early study of differential gene expression in spherules compared to mycelia was done using suppression subtractive hybridization [145]. This analysis identified SOWgp, PSP1 (a lipid transporter), an aldehyde reductase, a multidrug resistance transporter and an opsin-1 related gene as expressed in spherules but not mycelia. These findings have been confirmed by subsequent RNA-Seq studies.

Once the genomic sequences of *Coccidioides* spp. were available, experiments measuring large-scale gene expression levels in hyphae compared to spherules became feasible. This analysis is based on determining the number of RNA-Seq reads for a given predicted mRNA and requires careful statistical analysis. A transformation of the data using a negative binomial distribution and calculation of a false discovery rate, or a Benjamini–Hoch adjusted *p*-value is one method for comparison. Another issue is how to determine what fold change ratio is biologically meaningful. The usual values range from 2–4-fold; a higher fold change cut off is likely to result in fewer false-positive results but smaller changes in transcription still may be biologically important.

**Figure 11:**
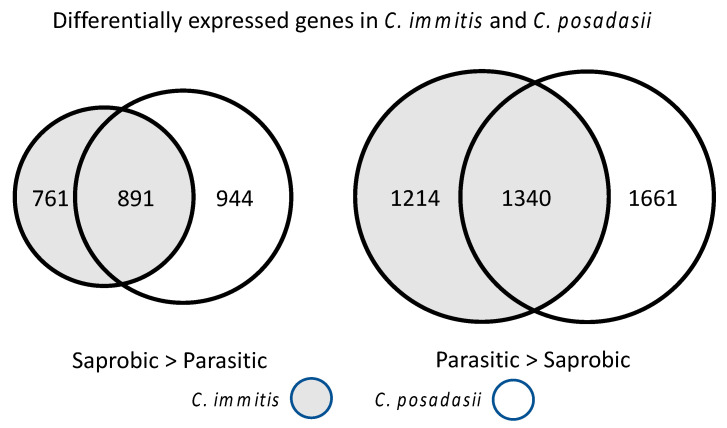
Comparison of differentially regulated genes in *C. immitis* and *C. posadasii.* Venn diagrams showing the number of genes commonly differentially regulated in the saprobic vs. parasitic growth phases of *C. immitis* and *C. posadasii*. The figure is from [15].

Several experiments comparing spherules grown in Converse medium to mycelia grown in glucose yeast extract medium have been reported. In addition to the growth phase, both the medium and the growth conditions (25 °C and room air for mycelia and 39 °C and 10% CO_2_ for spherules) are different between the two sets of samples. The initial study compared spherules and hyphae in *C. immitis* and *C. posadasii* [15]. They measured gene expression in spherules grown for four days. Three biological replicates were analyzed and ratios > 2-fold were considered to be significant. Thirteen percent of *C. immitis* and *C. posadasii* genes were preferentially expressed in hyphae and 19% of genes in the two species were preferentially expressed in spherules. There was a substantial amount of overlap between the differentially expressed genes in *C. immitis* and *C. posadasii* but about 50% of the genes that were up-regulated in spherules were only up-regulated in one species (Figure 11).

Genome Ontology (GO) classification is a structured vocabulary for describing characteristics of genes [146]. Genes with oxireductase GO terms were enriched in the set of genes that were preferentially expressed in spherules. In addition, some of the most up-regulated genes in spherules included Hsp30, Arp2/3, SOWgp, and several genes with no defined function. Several genes that had previously been found to be important in virulence were also up-regulated including 4-hydroxyl-phenyl pyruvate dioxygenase (4-HPPD), α-(1,3) glucanases, urease, and UGH. 4-HPPD has been found to be critical for yeast development in *Paracoccidioides brasiliensis* [147] and *Talaromyces (Penicillium) marneffei* [148]. Genes coding for cell surface proteins and secreted proteins were also up-regulated in spherules. Genes without a predicted function tended to be differentially regulated as well as those unique to *Coccidioides* spp. An experiment comparing spherules and mycelia using microarray technology showed similar results [71].

**Figure 12:**
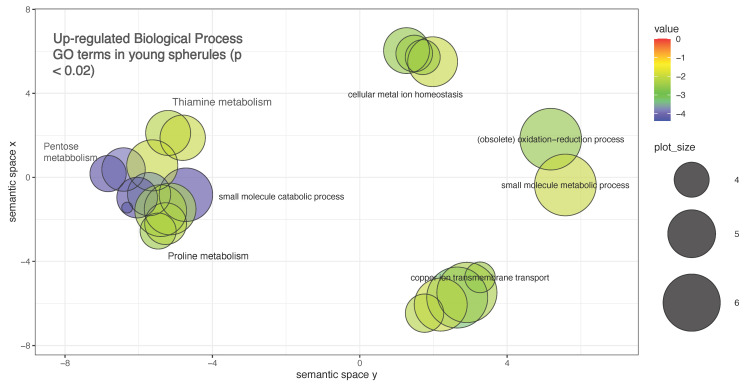
Biological GO terms enriched in spherules. Principle component analysis (PCA) plot of enriched GO terms of genes up-regulated in spherules, made using Revigo. The data for this figure is from [16].

Another experiment compared *C. immitis* hyphae to spherules after two days and eight days of spherule culture (young spherules and mature spherules) [16]. In this study a 4-fold ratio of counts was accepted as significant. There was relatively good correlation between the fold change results of this study and those obtained by Whiston although there were differences in the GO enrichment analysis. More than 20% of the genes were differentially expressed in hyphae compared to young or mature spherules. Expression of oxireductases, including a superoxide dismutase were up-regulated in spherules as was α-(1,3) glucan synthase. One gene that was highly up-regulated in this study, as well as several other studies, is opsin-1. As far as we know, *Coccidioides* is not light sensitive, and the function of this gene is unclear. One of the two genes coding for 4-HPPD was preferentially expressed in spherules. Analysis of GO terms in up-regulated genes in young spherules shows the enrichment for small molecule metabolic processes, copper and iron transport, and metal ion hemostasis (including iron)} (Figure 12). Iron import genes have been shown to be up-regulated in *Histoplasma capsulatum* yeast [149].

GO terms associated with membrane proteins were also enriched in spherules. Genes that are unique to *Coccidioides* spp. were preferentially expressed in spherules. A relatively small number of transposable elements were expressed at a low level and the majority of those were preferentially expressed in spherules. Most genes that were preferentially expressed in young spherules were also preferentially expressed in mature spherules, suggesting that many genes might be up-regulated for the whole life span of the spherule phase. One limitation of this study is that only one isolate of a species was investigated. As we accumulate more evidence using other methods, it becomes clear that there are significant differences between species and strains which might be biologically important.

Another technique to evaluate transcription is capped small RNA sequencing (csRNA-Seq). This technique captures newly initiated (RNA polymerase II) transcripts rather than the total amount of mRNA present in the cell, which is heavily influenced by RNA stability [150]. A study with *C. immitis* mycelia, day 2 spherules, and day 8 spherules showed drastic differences in transcription between spherules and mycelia [150]. A total of 11,728 newly transcribed isoforms were identified in mycelia and 19,771 isoforms were found in spherules. A total of 2240 (23%) of the newly transcribed RNAs were not found in the reference annotation. A large number of previously unrecognized transcriptional start sites were also identified in spherules. Many of these were promotor distal and initiated unstable RNAs. Comparing the newly transcribed transcripts in spherules and mycelia, the expression of 11,768 transcripts was up-regulated in spherules and the expression of 5,536 transcripts was down-regulated. These results are consistent with extensive remodeling of the genome during differentiation into spherules.

The up-stream regulatory motif that was most enriched in genes that were preferentially expressed in spherules was white-opaque switching regulator 1 (WOPR) motif, which is a motif that has been identified in a variety of fungi [151]. This DNA motif and the ryp1 transcription factor that binds to it are also important for phase transformation in *H. capsulatum* and *C. posadasii* [152,153]. A *C. immitis* ortholog of Ryp1, a transcription factor that drives transcription through the WOPR motif, was identified (CIMG_02671). This *C. immitis* gene was expressed in yeast and was shown to increase transcription of a reporter gene.

**Figure 13:**
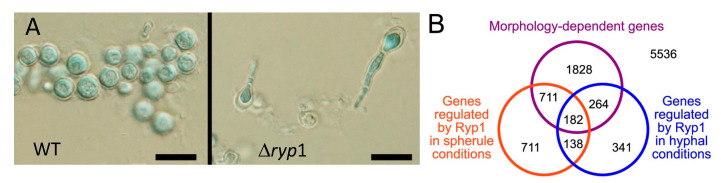
(**A**). Wildtype and Δ*ryp*1 *C. posadasii* grown in Converse medium in spherule conditions. Spherules form in the WT organism; only hyphae with polar swelling but no spherules are seen in the Δ*ryp*1 *C. posadasii* mutant. The bars represent 10 μM. (**B**). Gene expression in the Δ*ryp*1 mutant. Morphology-dependent genes; genes differentially expressed in spherules compared to mycelia Theses figures are from [153].

In addition to obtaining data about the transcriptome, an understanding of the overall transcription networks associated with conversion from arthroconidia to spherules would be incredibly helpful. In other fungi, “master regulators” have been found to influence the response to hyperosmotic stress [154], iron deprivation [155], and differentiation from mold to yeast (Drk1) [156]. A recent paper found an important master regulator in *C. posadasii* [153]. Ryp1 is an important regulator that influences mycelial to yeast differentiation in *H. capsulatum* [152]. To determine the role of this gene in *C. posadasii* a *Ryp1* knock-out mutant was made. The deletion mutant did not grow as well as wildtype in the mycelial phase, but the more striking phenotype is that it did not form any spherules under spherule growth conditions (elevated temperature, elevated CO_2_ in Converse medium) in vitro. A small number of organisms grew as hyphae with polar swelling (Figure 13). The mutant was avirulent in mice and no viable organisms could be recovered from the lung or spleen. Therefore, this transcription factor influenced the expression of many of the genes coding for differentiation into spherules and pathogenicity.

The effect of Ryp1 on gene expression in spherules and hyphae was also studied by RNA-Seq. A total of 1742 transcripts were differentially expressed in the *Δryp1* deletion compared to wildtype under spherule growth conditions, and in mycelial growth conditions 925 transcripts were differentially expressed. Many but not all the differentially expressed genes were also differentially expressed in spherule/mycelium comparisons and these are of great interest.

Another study also explored the transcriptome of *Coccidioides* spp. mutants that do not form normal spherules [157]. A disruption mutant of chitinase 2 and 3 (*cts*2, *cts*3) and D-arabinotol-2-dehydrogenase in *C. posadasii* forms pre-segmented spherules but does not endosporulate and is not pathogenic [102] (see Section 9.1). The relative transcription (spherules/hyphae) of genes in this mutant was compared to the relative transcription of the wildtype strain. A total of 222 genes were up-regulated in wildtype spherules but not the mutant. Enriched GO terms for these genes included proton export, sulfate transport, nitrate metabolism, response to oxidative stress, and oxidation/reduction processes. This suggests that genes coding for ion transport and response to oxidative stress functions were required for formation of endosporulating spherules. Half of the differentially expressed genes in the wildtype but not the mutant, were annotated as hypothetical proteins. Eight of the differentially expressed genes only had orthologs in *Coccidioides* spp., so they were unique, or orphan genes, as other studies have found [15,16]. Of the seven very highly up-regulated wildtype spherule genes that were not up-regulated in the mutant, four were also up-regulated in *C. immitis* spherules [16]. There were 19 genes that were up-regulated in wildtype spherules but down-regulated in the mutant spherules. The enriched GO terms included iron acquisition, metal homeostasis and response to oxidative stress. A total of 18 genes were down-regulated in wildtype spherules but up-regulated in the mutant. These genes were involved in chitin remodeling and pheromone sensing. This study suggests that studying the transcriptome of mutants that cannot form normal spherules is a useful tool to predict genes that are required for phase conversion.

### 8.2. Comparison of Mycelia and Spherules by Proteomics

Lake and his co-workers have evaluated the proteomics of *Coccidioides* spp. Spherules in several ways. One study examined the proteins in spherulin [158]. Spherulin contained 1390 proteins and a number of these were glycosylation enzymes. Spherules could be detected by wheat germ agglutinin (WGA) lectin and *Griffonia simplificonia* lectin II (GSL-II) staining in vivo and both these lectins bound to spherule proteins in an ELISA. WGA binds to N-acetylglucosamine dimers and trimers; GSL-II binds to N-acetylglucosamine residues on the non-reducing ends of oligosaccharides. A total of 274 proteins were obtained by lectin affinity purification. The five most highly expressed glycoproteins were 5-methyltetrahydropteroyl-triglutamate-homocysteine methyltransferase (CPSG_03208), malate dehydrogenase (CPAG_07192), fructose biphosphate aldolase (CPAG_09270), enolase (CPAG_04681), and 3-isopropylmalate dehydrogenase (CPAG_08709). Other relatively highly expressed glycoproteins included heat shock protein 90 (CPAG_06539) and complement fixation-chitinase (CPSG_08657).

A study of the abundance of glycosylation enzymes in mycelia and spherules (both in vitro and in vivo) was also done [77]. Seventy-one percentof the *Coccidioides* spp. glycosylation enzyme family proteins predicted from the genome annotation were detected. Spherules were grown in both Converse and RPMI media with a variety of modifications; a total of six different culture conditions were evaluated. A total of 111 glycosylation enzymes were identified in at least one of the culture systems and 47 enzymes were detected in infected human lungs. Choline oxidase, β-glucosidase 4, NADP-dependent leukotriene B4, and 12-hydroxydehrogenase were more abundant in spherules than in mycelia whereas endochitinase, β-hexosaminidase 1, α-mannosidase, mannosyl-oligosaccharide α1,2-mannosidase, and a β-1,3-glucanase were more abundant in mycelia than spherules. The differential expression of β-hexosamidase 1 and β-1,3-glucanase was also seen in one of the RNA-Seq studies but the other proteomic observations were not [16].

Normal human lung tissue and lung tissue in patients infected with *Coccidioides* spp. were also analyzed for glycan content and composition. Infected lung tissue contained 292 unique glycans and only one third of them were previously known [77]. The authors suggest these glycans might be useful for the diagnosis of human disease.

**Figure 14:**
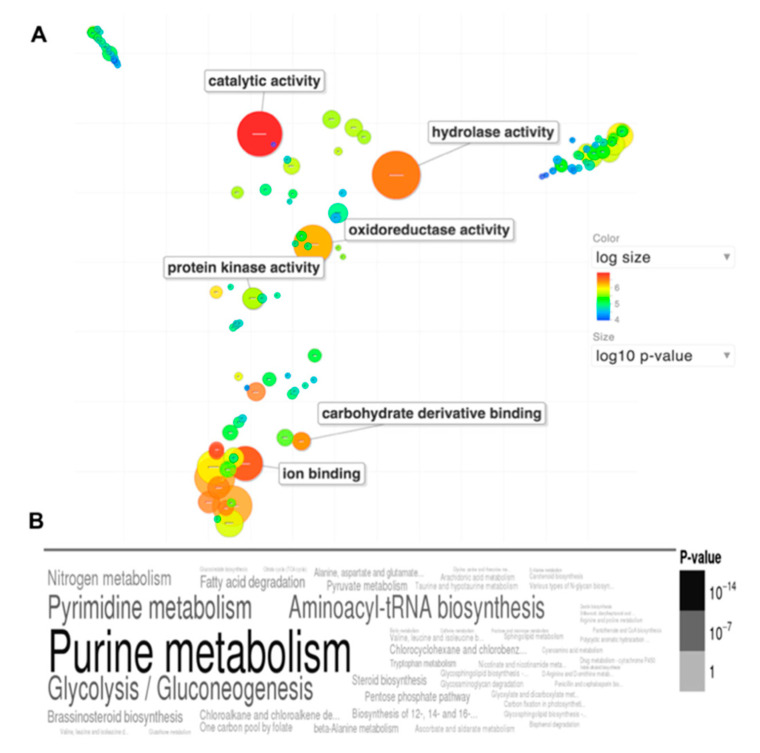
Gene ontology enrichment analysis of the 27 biomarker candidates indicates enriched hydrolase activity and increased purine and carbohydrate metabolism functions. (**A**) Scatterplot of gene ontology enrichment of molecular functions, with size of the circles is proportional to the significance of enrichment. (**B**) Word cloud of enriched KEGG metabolic pathways. Size of the words indicates the significance of enrichment. Figures were produced using Revigo and GOSummaries. The figure is from [77,78].

Another study examined expression of *Coccidioides* spp. proteins in infected human lungs [78]. Laser capture micro-dissection was used to sample areas containing spherules and the proteins were extracted and analyzed. Of the 100 most abundant proteins, 27 were found in *Coccidioides* spp. but not humans; four of those proteins were found only in *Coccidioides* spp. and not in other pathogenic fungi (Figure 14).

GO analysis of the 27 proteins that were highly expressed in human lungs revealed increases in hydrolase and oxireductase activities (Figure 14). KEGG metabolic pathways were enriched for purine metabolism and gluconeogenesis. About 50% of the proteins contained a glycosylation site and 25% had a predicted signal peptide. The abundance of these 27 proteins in tissue was compared to the abundance in spherules cultured in vitro and six proteins were significantly more abundant in tissue. These were hsp20/alpha crystallin family protein, peroxisomal matrix protein, cytochrome c oxidase polypeptide VI, and uncharacterized proteins CISG_02340, CIMG_09001, and CIMG_05576. These same proteins were preferentially expressed in spherules compared to mycelia in vitro.

The level of expression of these proteins in vitro also depended on the spherule growth conditions. Of the 27 proteins, 96% were expressed in spherules grown in RPMI + Survanta (a pulmonary surfactant), but smaller numbers were found in standard Converse medium + Tamnol-N or RPMI + fetal calf serum. Thus, spherules grown in different conditions differ in the expression of some proteins and a more comprehensive study of gene and protein expression under these conditions is clearly warranted.

### 8.3. Comparison of the Metabolomics of Mycelia and Spherules

The biology of spherule development has also been investigated by characterizing the volatile organic compounds produced by mycelia and spherules. There were dramatic differences in the volatile organic compounds (VOC) produced by the two forms of the fungus [159]. The VOC produced by a number of strains of *C. immitis* and *C. posadasii* were similar, but spherules and mycelia had very different profiles.

The VOC of mycelial and spherule cultures of six *C. posadasii* and six *C. immitis* isolates were determined. Aromatic hydrocarbon 1-methyl-3-(1-methylethyl)-benzene and the aldehyde hexanal VOCs are more frequently found in mycelial cultures, while the alcohols 2-(2-butoxyethoxy)-ethanol and 2-hexen-1-ol, the ketone cyclohexanone, and the heteroaromatic 2,4,6-trimethylpyridine were associated with spherule cultures. There were no VOCs that were significantly different in relative abundance between *C. posadasii* and *C. immitis*. However, there was a good deal of variation from isolate to isolate. More than 250 spherule volatiles were detected, but only 13% of these were detected in at least two thirds of all spherule cultures. One third of these VOCs are also significantly more abundant in spherule than in mycelial cultures. A principal-component analysis (PCA), using the 78 cultures (12 isolates, from mycelia and spherules for each isolate and 3 replicates) and controls showed that the volatilomes cluster by life cycle rather than species. A hierarchical clustering analysis confirmed the difference between spherules and mycelia.

Although there are clear differences between the VOC produced by spherules and mycelia, the functional importance of these results for fungal biology and pathogenesis are difficult to determine. Understanding the enzymatic pathways that produce these products would be necessary for making genetic deletion mutants to test the importance of these differences. Another approach would be to compare the metabolome of mutants that don’t form mature spherules to normal spherules and mycelia.

### 8.4. Integrating Data about Mycelia and Spherules

It is difficult to draw overall conclusions from all the “-omic” studies. The RNA-Seq studies of *Coccidioides* spp. yield similar but not identical results and authors have chosen to emphasize different aspects of the data. That is a consequence of having so much data to analyze and the need to focus on selected parts of the data for an overall conclusion in a publication. RNA-Seq studies with more stages of differentiation (very early spherules, a time series of spherules and endospores) would be very useful. RNA-Seq studies in the WA-2 *C. immitis* strain and other sequenced *C. posadasii* strains would also be useful as would further studies of spherule differentiation using other conditions such as RPMI with fetal calf serum or Survanta. RNA-Seq study of spherules in vivo would be incredibly useful. Finally, cataloging the results in a searchable database is critical for drawing useful information about consistent results.

However, it is important to realize the limitations of RNA-Seq studies. It is difficult to know what effect many genes have on a given genetic or metabolic pathways and many genes may have overlapping functions. When different strains, species or growth conditions are used and experiments are done in different laboratories, it is hard to aggregate the data in a meaningful way. A more fundamental problem is that genes that are required for differentiation into spherules do not necessarily need to be expressed at higher levels as spherule differentiation occurs. All these studies can accomplish is establishing an association between a biological process and relative quantitative gene expression. To establish the importance of a gene it must be deleted or inhibited.

Proteomics is another approach to identifying biologically important molecules. Since proteins, rather than mRNA, are the gene products, it would seem that this approach would be preferable to RNA-Seq. However, only about 10% of the proteins predicted by genome annotation were detected in coccidioidin and spherulin [91] and comparisons of spherules to hyphae and were done using a small number of the most common proteins even though there is no guarantee that common proteins are the most important for differentiation from mycelia to spherules.

The final goal of all these studies is determining what genes and metabolic pathways are important for the life and pathogenicity of the spherule. The most definitive proof of the biological importance of a gene is to make a deletion mutant and characterize its biology. Making targeted deletions of genes to prove their biological effects is a costly and time-consuming effort, so careful prioritization of genes based on the integration of all available data is critical.

## 9. Genetic Manipulation

Protoplast-mediated transformation [160] and *Agrobacterium*-mediated transformation are two approaches [161] to making targeted gene deletions in *C. posadasii*. For both approaches, a plasmid is made with the flanking regions of the gene of interest disrupted by a selectable marker, usually the hygromycin resistance gene. In the transformation method, protoplasts are transformed with linear DNA constructs using polyethylene glycol and calcium ions [101]. Unfortunately, making viable protoplasts is technically difficult. Once the protoplasts have recuperated, the recombinant organisms are selected. A detailed protocol has been published [162]. This approach has been used for anumber of gene disruption studies. Hopefully newer techniques for genetic manipulation, such as CRISPR technology will soon be avaible for *Coccidioides* spp.

For *Agrobacterium*-mediated transformation, the plasmid is inserted into T-DNA and transformed into *Agrobacterium* [163]. A major advantage of this approach is that there is no need to make protoplasts. The *Agrobacterium* are used to transfer the plasmid into *C. posadasii,* and the resistant clones are selected by growth on hygromycin. When this technique was used to delete the CPS1 gene, 7/27 clones were identified that were homologous gene replacements [164].

Both techniques require multiple rounds of subculturing because *Coccidioides* spp. have multiple nuclei per cell. In addition, screening by PCR and DNA sequencing the antibiotic resistant colonies is critical because a significant number of ectopic insertions occur. This approach should potentially work as well in *C. immitis,* but successful transformation has not been reported in that species.

### 9.1. Engineered Avirulent Mutants

Random mutagenesis of the *C. immitis* RS strain by UV irradiation resulted in a number of mutants [165]. Two of these were temperature-sensitive and auxotrophic. These mutants formed spherules but were avirulent and were effective as live vaccines [165,166,167]. These strains have not been genetically characterized and have been lost.

Genetically engineered attenuated strains have also been made. *C. posadasii* contains a single 1,3-β-glucan synthase gene [168]. This gene is required for viability, so a viable deletion mutant strain could not be made, as has been seen in other fungi.

Deletion of two chitinase genes (*CTS*2 and *CTS*3) and the *ARD1* gene in *C. posadasii* C735 using the protoplast-mediated transformation method resulted in a mutant that grew normally in the mycelial phase but formed sterile spherules [102]. The spherules did not form endospores even after prolonged culture in Converse medium. The *cts*2*/ard*1*/cts*3 deletion strain was avirulent even at a very high inoculum in susceptible mouse strains, while strains with a single cts1 deletion or the cts2/ard1 deletion were pathogenic. The *cts*2*/ard*1*/cts*3 deletion strain was also a highly effective live vaccine. The chitin synthase 5 gene has also been deleted in *C. posadasii* and that strain, Δ*CHS*5, is avirulent and has been classified as a BSL-2 organism [169]. As far as we can determine, there is no literature describing this mutant further.

Another avirulent *C. posadasii* mutant strain has been made by deleting the *C. posadasii* homolog of the *CPS*1 gene [164]. The *CPS*1 gene plays an important role in the pathogenicity of the maize pathogen *Cochliobolus heterostrophus*, although the mechanism of that activity is not known. The mutant strain grew slightly slower than wildtype at 25 °C in the mycelial phase and formed only 10% as many arthroconidia. A transcription study comparing mutant to wildtype (both in the spherule phase) found that only 33 genes were differentially regulated with 17 down-regulated in the mutant and 16 up-regulated. Of the 17 down-regulated genes, 5 were hypothetical, 1 was a glyoxylate reductase, 2 were stress response genes, and 1 was a serine/threonine protein kinase. When grown in Converse medium, the mutant formed spherules that were somewhat smaller than wildtype spherules, but it is not clear whether endosporulation occurred. However, this mutant was completely avirulent in genetically susceptible but immunocompetent mice, even when an inoculum of 4000 arthroconidia was used. In highly immunocompromised mice one animal had significant fungal growth. Histologic studies early in infection with a very high inoculum showed occasional spherules lacking endospores that were surrounded by neutrophils. In addition to being non-pathogenic, the cps1 deletion mutant was also an effective vaccine.

The Δryp1 mutant is also avirulent (see Section 8.1) [153]. This mutant differs from the two described above because it does not form spherules in vitro. Interestingly, unlike other avirulent *C. posadasii* mutant strains, immunization with the Δ*ryp*1 mutant did not confer resistance to infection, possibly due to the lack of host exposure to spherule-specific antigens due to the Δ*ryp*1 defect in spherule morphology in vivo.

### 9.2. Pathogenicity of Naturally Occurring Coccidioides Spp. Strains

Not all *Coccidioides* spp. strains are equally capable of causing disease in animals [170,171]. *C. immitis* H538.4, which was isolated from the soil in the San Joaquin Valley was not lethal in mice, although it caused systemic infection. Histologic examination showed that the spherules only formed a small number of large endospores, so a block in endospore development seemed a likely reason for the decrease in pathogenicity. The genome of this strain was sequenced, but, unfortunately, it has been lost and is not available for further study. Other strains, including *C. posadasii* RMSCC 1038 and RMSCC 3700 are less virulent than most isolates [172]. The pathogenicity of *C. posadasii* RMSCC 1038 has recently been studied in susceptible and resistant mouse strains [173]. Infection with this strain caused relatively indolent disease in genetically susceptible mice compared to *C. posadasii* strain Silviera, but ultimately the mice died over the course of 6–10 weeks. In genetically resistant mice, there was no increase in the number of organisms in the lungs over the four months after infection and the numbers in the spleen decreased. In an experiment carried out for 9 months in resistant mice, the lung and splenic fungal burdens decreased after four months of infection. The mice gained weight throughout this period and none of them died. The reasons for the difference between this strain and highly virulent *C. posadasii* strains (such as Silviera) are not known. Using the *C. posadasii* RMSCC 1038 strain for challenge in mice causes a clinical syndrome that is similar to a typical disease in human beings and should allow characterization of the immune response over time. These studies indicate that there are dramatic differences in the pathogenicity of different strains but a systematic study of the pathogenicity of a large number of isolates (including soil isolates) has not been done.

## 10. Strain Availability

A total of 17 strains are currently available from a central repository, including two mutant strains derived from *C. posadasii* C735. This can be accessed at bei Resources (https://www.beiresources.org/Catalog.aspx?f_instockflag=In+Stock%23~%23Temporarily+Out+of+Stock&q=Coccidioides) (accessed on 10 August 2022). A much larger number of strains has been used over the years in a variety of laboratories. There are definitely a relatively large number of other strains that are currently maintained, and sharing these, probably through bei Resources, would be very useful for *Coccidioides* spp. research in general.

## 11. Conclusions

*Coccidioides* spp. are fascinating organisms for a variety of reasons. They are one of a select group of dimorphic environmental fungi that cause human and animal disease. The parasitic phase of the organism, the spherule-endospore cycle, is not seen in any other fungus. They are able to live in a dry, harsh natural environment. Although the organism is endemic in a limited geographic region, the numbers of infections in those regions have increased dramatically over the past few years and the endemic area may be expanding. The infection they cause can be serious and, in some instances, fatal, even in immunocompetent people. There are many questions about these organisms that remain unresolved. A few specific questions include the amount of biological variability between species and within strains, characterization of the genetic and biochemical pathways that cause differentiation to spherules, identification of virulence genes, identification of effective proteins for vaccines, development of new diagnostic tests, and understanding the factors that control the growth of the organism in the environment. Answering these questions should provide information that will be helpful for understanding coccidioidomycosis and ultimately improving medical management of this important infection.

The number of investigators studying this organism has grown steadily and the techniques and methodologies for investigation have become much more sophisticated. In addition, funding for the study of *Coccidioides* spp. and coccidioidomycosis has grown substantially. Given this, we are hopeful that our understanding of this fascinating fungus and the disease that it causes will continue to improve rapidly.

## Figures and Tables

**Table 1:** Pathogenicity of some *C. posadasii* deletion mutants.

Experiment	Deletion	% Mortality	CFU/Lung (log_10_)
1	None	100	NA
	SOWgp	40	NA
2	None	100	7.2
	urease	30	3.6
	ugh	25	4.4
	urease/ugh	10	2.1

Results of experiments with *C. posadasii* deletion mutants. CFU, colony forming unit. NA, not applicable. The data is from [136,139].

## Data Availability

Not applicable.
